# Thromboembolic Events After Vitamin K Antagonist Reversal With 4-Factor Prothrombin Complex Concentrate: Exploratory Analyses of Two Randomized, Plasma-Controlled Studies

**DOI:** 10.1016/j.annemergmed.2015.04.036

**Published:** 2015-06-17

**Authors:** Truman J. Milling, Majed A. Refaai, Joshua N. Goldstein, Astrid Schneider, Laurel Omert, Amy Harman, Martin L. Lee, Ravi Sarode

**Affiliations:** Seton/UT Southwestern Clinical Research Institute of Austin, University Medical Center at Brackenridge, Dell Children’s Medical Center, Austin, TX; University of Rochester Medical Center, Rochester, NY; Massachusetts General Hospital, Boston, MA; CSL Behring GmbH, Marburg, Germany; CSL Behring LLC, King of Prussia, PA; CSL Behring LLC, King of Prussia, PA; UCLA School of Public Health, CA; UT Southwestern Medical Center, Dallas, TX.

## Abstract

**Study objective:**

We evaluated thromboembolic events after vitamin K antagonist reversal in post hoc analyses of pooled data from 2 randomized trials comparing 4-factor prothrombin complex concentrate (4F-PCC) (Beriplex/Kcentra) with plasma.

**Methods:**

Unblinded investigators identified thromboembolic events, using standardized terms (such as “myocardial infarction,” “deep vein thrombosis,” “pulmonary embolism,” and “ischemic stroke”). A blinded safety adjudication board reviewed serious thromboembolic events, as well as those referred by an independent unblinded data and safety monitoring board. We descriptively compared thromboembolic event and patient characteristics between treatment groups and included detailed patient-level outcome descriptions. We did not power the trials to assess safety.

**Results:**

We enrolled 388 patients (4F-PCC: n=191; plasma: n=197) in the trials. Thromboembolic events occurred in 14 of 191 patients (7.3%) in the 4F-PCC group and 14 of 197 (7.1%) in the plasma group (risk difference 0.2%; 95% confidence interval −5.5% to 6.0%). Investigators reported serious thromboembolic events in 16 patients (4F-PCC: n=8; plasma: n=8); the data and safety monitoring board referred 2 additional myocardial ischemia events (plasma group) to the safety adjudication board for review. The safety adjudication board judged serious thromboembolic events in 10 patients (4F-PCC: n=4; plasma: n=6) as possibly treatment related. There were 8 vascular thromboembolic events in the 4F-PCC group versus 4 in the plasma group, and 1 versus 6 cardiac events, respectively. Among patients with thromboembolic events, 3 deaths occurred in each treatment group. All-cause mortality for the pooled population was 13 per group. We observed no relationship between thromboembolic event occurrence and factor levels transiently above the upper limit of normal; there were no notable differences in median factor or proteins C and S levels up to 24 hours postinfusion start in patients with and without thromboembolic events.

**Conclusion:**

The incidence of thromboembolic events after vitamin K antagonist reversal with 4F-PCC or plasma was similar and independent of coagulation factor levels; small differences in the number of thromboembolic event subtypes were observed between treatment groups. [Ann Emerg Med. 2016;67:96-105.]

## INTRODUCTION

### Background

Health care providers prescribe warfarin, a vitamin K antagonist, to more than 3.4 million patients annually in the United States for various prothrombotic conditions.^1^ The Food and Drug Administration describes warfarin as among the top 10 drugs with the largest number of serious adverse drug reactions submitted during the 1990s and 2000s.^2^

Vitamin K antagonists reduce the risk of thromboembolic events in patients with prothrombotic conditions by inhibiting the synthesis of functional vitamin K–dependent clotting factors (factor [F] II, VII, IX, and X) and proteins C and S. Patients receiving vitamin K antagonists who are actively bleeding or in need of an urgent surgical procedure need rapid reversal of anticoagulation by prompt replenishment of vitamin K–dependent clotting factors. In the current literature and guidelines, a combination therapy of vitamin K and either plasma or prothrombin complex concentrate is recommended for acute reversal of oral anticoagulation.^3–8^

The efficacy of plasma for urgent vitamin K antagonist reversal has not been established, and although it is less expensive per unit than other reversal methods, it has several drawbacks. Plasma may be a carrier of infective agents, requires ABO blood typing and thawing before use, is subject to relatively long infusion times, and can be associated with severe adverse outcomes, including transfusion-associated circulatory overload and transfusion-related acute lung injury.^3,9-12^ In contrast, prothrombin complex concentrates do not require cross-matching or thawing, undergo a number of viral inactivation and elimination steps during their manufacturing process to minimize risk of viral transmission, do not pose a risk of transfusion-related acute lung injury, and can be infused in 15 to 30 minutes.^3^

The use of prothrombin complex concentrates for anticoagulation reversal may be associated with thromboembolic complications; however, most of the studies on this subject are small retrospective case studies that lack a comparison group of other reversal agents, such as plasma.^13^ It is difficult to differentiate the risk of the therapy used for anticoagulant reversal from the underlying risk of thrombosis. Recently, 2 phase 3b randomized controlled trials comparing the efficacy and safety profile of a nonactivated 4-factor prothrombin complex concentrate (4F-PCC) (Beriplex; CSL Behring, Marburg, Germany; known as Kcentra in the United States) versus plasma in vitamin K antagonist–treated patients requiring urgent anticoagulation reversal (owing to acute major bleeding^14^ or before emergency surgery^15^) were completed. The similarity in patient demographics, treatment regimens, and thromboembolic safety outcomes for the 2 studies may permit the pooled analysis of safety data, providing a larger data set to compare the safety profiles of the 2 interventions. Therefore, we report the complete set of thromboembolic events with patient-level narratives and findings in addition to aggregate data.

### Importance

The analysis provides a unique opportunity to evaluate the safety profile of a 4F-PCC in comparison with plasma in terms of thromboembolic events, and deaths in patients with those events. Safety data extend to 45 days postinfusion, enabling the evaluation of the timing and characterization of thromboembolic events far beyond the immediate postinfusion period.

### Goals of This Investigation

Our objective was to evaluate and compare the frequency and characteristics of thromboembolic events that occurred during 2 randomized controlled trials that compared a 4F-PCC with plasma for urgent vitamin K antagonist reversal.

## MATERIALS AND METHODS

### Study Design and Setting

We performed a secondary post hoc analysis on safety data collected during 2 prospective, randomized, open-label, active-controlled, noninferiority, phase 3b trials.^14,15^ Patients receiving vitamin K antagonists and presenting with acute major bleeding (NCT00708435) or in need of urgent surgical or invasive procedures (NCT00803101) were randomized to receive 4F-PCC (containing vitamin K–dependent factors plus proteins C and S) or plasma.

The population evaluated in this analysis (pooled safety population) comprised patients from both studies who received any portion of study product.^14, 15^ Each patient received his or her assigned study treatment on day 1, dosed according to baseline international normalized ratio and body weight (Table E1, available online at http://www.annemergmed.com)^14, 15^; all patients were also to receive vitamin K. Resumption of anticoagulant therapy was to be considered after the stabilization of the acute bleeding event or as soon after the procedure as medically appropriate. Study staff drew blood samples for determination of levels of vitamin K–dependent factors and proteins C and S before study product infusion and 0.5, 1, 3, 6, 12, and 24 hours after start of infusion.

Primary study investigators recorded adverse events and concomitant medications at every point up to day 10 (visit window days 7 to 11), and serious adverse events up to day 45 (visit window days 43 to 51). An independent data and safety monitoring board reviewed unblinded data to assess patient safety and also assessed serious adverse events and deaths. A blinded, independent safety adjudication board reviewed serious adverse events of interest to the data and safety monitoring board (including thromboembolic events) for case confirmation and relatedness to study product administration.

Any potential thromboembolic event was first evaluated for seriousness and relatedness by the unblinded investigator. The unblinded independent data and safety monitoring board (which reviewed all adverse events) could also refer suspected thromboembolic events to the safety adjudication board for review. The blinded safety adjudication board evaluated any serious thromboembolic events or data and safety monitoring board–referred events for relatedness, meaning any thromboembolic event had at least 1, and possibly 2, independent evaluations for relatedness. See Appendix E1 (available online at http://www.annemergmed.com) for further explanation of standard terms and processes.

We have previously described additional details of the study designs, patient randomization, treatment allocation, inclusion and exclusion criteria, and methods, as well as efficacy and top-line safety results.^14, 15^

CSL Behring sponsored the studies, which were performed in accordance with local ethics regulations, the International Conference on Harmonization Good Clinical Practice guidelines, and the Declaration of Helsinki (1996). Investigators obtained written informed consent from all patients before participation in any study procedure or assessment.

### Primary Data Analysis

We used data from the trial databases to generate a report for all variables of interest for this analysis from all patients in the pooled safety population. Variables of interest included reason for oral vitamin K antagonist therapy, type of thromboembolic event, relationship to treatment, additional thromboembolic risk factors, and time from treatment with study product to event onset. We also evaluated the relationship between coagulation factor and protein levels and event occurrence.

Because the trials were not powered to support any testing of this subset of adverse event cases, we treated the testing of data as exploratory and reported all differences with ranges and confidence intervals. We determined the confidence interval for the risk difference by the Wilson method, using the correction for continuity.

We analyzed coagulation factor and protein levels at each point with a 2-tailed Wilcoxon rank sum test for between-group comparisons and a signed rank test for within-group changes from baseline.

## RESULTS

### Characteristics of Study Subjects

The pooled safety population comprised 388 patients (4F-PCC: n=191; plasma: n=197). The median prothrombin complex concentrate dose was 25 IU/kg (range 15.5 to 50.5 IU/kg), with a median volume of 90 mL (range 48 to 230 mL) and median infusion time of 17 minutes (range 7 to 288 minutes). The median plasma dose was 10 mL/kg (range 3.9 to 17.7 mL/kg), median volume 800 mL (range 353 to 1,525 mL), and median infusion time 120 minutes (range 22 to 928 minutes). For patients who experienced thromboembolic events, the demographics, disease characteristics (excluding vitamin K antagonist reversal indication), and patient disposition were similar between the 2 studies (Table 1).^14, 15^

### Main Results

In total, 28 of 388 patients (7.2%) in this analysis population experienced greater than or equal to 1 thromboembolic event (4F-PCC: 14/191 [7.3%]; plasma: 14/197 [7.1%]), with a risk difference of 0.2% (95% confidence interval −5.5% to 6.0%). The breakdown by study was 6 and 8 4F-PCC–treated patients with events in the surgical and bleeding study, respectively, and 7 plasma-treated patients with events in each study, for a total of 14 patients with events in each treatment group in the combined analysis.

Three patients experienced a second thromboembolic event, meaning that the total number of events considered in this analysis was 31 (4F-PCC: n=16 in 14 patients; plasma: n=15 in 14 patients) (Table 2). Site investigators reported 17 thromboembolic events as serious (predefined standardized terms; 4F-PCC: n=9; plasma: n=8), and the independent data and safety monitoring board referred 2 suspected thromboembolic events (myocardial ischemia, plasma group) to the safety adjudication board for review. The blinded safety adjudication board subsequently confirmed 16 of these 19 events as thromboembolic (4F-PCC: n=7; plasma: n=9).

Eleven of 16 thromboembolic events (68.8%) in the 4F-PCC group occurred more than 7 days after infusion, clustering in the second week (Figure). In comparison, the majority of events in the plasma group (11/15 [73.3%]) occurred within 7 days of infusion, with 7 events (46.7%) occurring on the day of infusion compared with 3 (18.8%) in the 4F-PCC group (Figure).

Additional event characteristics are reported in Table 3. There were 5 nervous system events in 5 patients in each treatment group. Eight vascular events (in 7 patients) occurred in the 4F-PCC group compared with 4 events in 4 patients in the plasma group. Of these vascular events, 4 deep venous thrombosis events occurred in 3 patients in the 4F-PCC group (patient K11 and K12; event count also includes the basilic vein clot due to peripherally inserted central catheter in patient K6), and 1 deep venous thrombosis (patient P7) and 1 pulmonary embolism (patient P12) were reported in the plasma group (Tables 2 and 3). One cardiac event (myocardial infarction) occurred in the 4F-PCC event group and 6 cardiac events (2 myocardial ischemia events [referred by the data and safety monitoring board] and 4 myocardial infarctions/acute myocardial infarctions) occurred in the plasma event group (Table 3). Causality by event type is summarized in Table E2, available online at http://www.annemergmed.com.

Patients with thromboembolic events had a variety of underlying risk factors, the most frequent being hypertension, atrial fibrillation, coronary artery disease, and congestive heart failure (Table E3, available online at http://www.annemergmed.com). The majority of patients with reported events (19/28 [67.9%]) were receiving vitamin K antagonist therapy for arrhythmias (Table 1). The predominant type of major bleeding was gastrointestinal or other nonvisible hemorrhage (9/15 [60.0%] patients with events in the acute bleeding study), whereas the predominant type of surgery or procedure was major orthopedic procedures (5/13 [38.5%] patients with events in the surgery study) (Table 1).

Resumption of anticoagulation was at the discretion of the treating physician, and the majority of patients in this subset had not resumed anticoagulation therapy at the event despite the presence of multiple risk factors for thromboembolism (only 3 patients in the 4F-PCC event group and 4 in the plasma event group were receiving anticoagulant therapy at the event) (Table 1). In addition to the nonresumption of anticoagulant therapy, we observed that 9 of 14 patients (64.3%) in the 4F-PCC event group and 6 of 14 (42.9%) in the plasma event group had an event consistent with the original indication for anticoagulation (Table E3, available online at http://www.annemergmed.com).

Results indicate that the risk of thromboembolic events was not proportional to dose and that dose was not associated with the development of an event in either group. In the 4F-PCC event group, 7 of 14 patients (50.0%) received the dose at 25 IU/kg, 3 of 14 (21.4%) received it at 35 IU/kg, and 4 of 14 (28.6%) received it at 50 IU/kg (Table E3, available online at http://www.annemergmed.com). In the plasma event group, 5 of 14 patients (35.7%) received an actual dose of less than 10 mL/kg, 8 of 14 (57.1%) received 10 to 15 mL/kg, and 1 of 14 (7.1%) received greater than 15 mL/kg (Table E3, available online at http://www.annemergmed.com).

We summarize other characteristics for patients with and without events, as well as for the entire pooled safety population, in Table 4. A slightly larger proportion of patients with events were aged 70 years or older compared with those who did not experience an event (64.3% versus 54.4%, respectively). A larger proportion of patients with events had been prescribed vitamin K antagonist for arrhythmia (67.9% versus 48.6%, respectively) or a remote thromboembolic event (28.6% versus 17.5%, respectively) compared with patients without an event.

The majority of patients who experienced an event recovered without sequelae. As has been previously reported, the 45-day all-cause mortality was 13 patients per group for the pooled safety population (4F-PCC: 10 and 3 patients in the bleeding and surgery study, respectively; plasma: 5 and 8 patients, respectively).^14, 15^ Among patients who experienced a thromboembolic event, 3 from the 4F-PCC event group (K1, K13, and K14) and 3 from the plasma event group (P8, P9, and P12) died during the study (Table 2). The blinded safety adjudication board did not adjudicate any of the deaths in the 4F-PCC event group as related to study product. Patient K1 (aged 66 years) experienced a thrombosis in device (fistula clot/thrombosis) on day 1 but died of sepsis on day 26. Patient K13 (aged 88 years) died of unknown causes on day 38; investigators attributed the death to a myocardial infarction, leading the safety adjudication board to assess it as a possible thromboembolic event. The safety adjudication board did not categorize the case as an event owing to its timing, lack of compatible medical history, and unknown cause of death; hence, the board did not assess the causality for the myocardial infarction and death. The death of patient K14 (aged 89 years) on day 46 followed an ischemic stroke on day 43. Although the investigator considered the ischemic stroke possibly related to study product, the safety adjudication board did not agree owing to the timing of the event, the lack of anticoagulation in a patient with atrial fibrillation, and the patient’s age and comorbidities.

In the plasma group, patient P8 (aged 85 years) died of congestive heart failure on day 13 but had experienced a stroke (reported as embolic cerebral infarction) approximately 34 hours after plasma infusion. The investigator reported contributing illnesses as severe tricuspid regurgitation, pulmonary hypertension, and chronic obstructive pulmonary disease; the safety adjudication board considered the stroke to be possibly related to study product, citing the small interval between the plasma administration and the event. The death of patient P9 (aged 61 years), admitted for cardiac catheterization and aortic valve replacement, on day 8 followed a myocardial infarction the same day and multiple coronary occlusions and cardiac events in the days leading up to his death. The safety adjudication board considered both the myocardial infarction and the death to be possibly related to study product. The death of patient P12 (aged 92 years) on day 16 was likely due to profound anemia resulting in congestive heart failure in an already compromised cardiac patient, and the safety adjudication board did not consider it related to study product.

**Figure E1.**
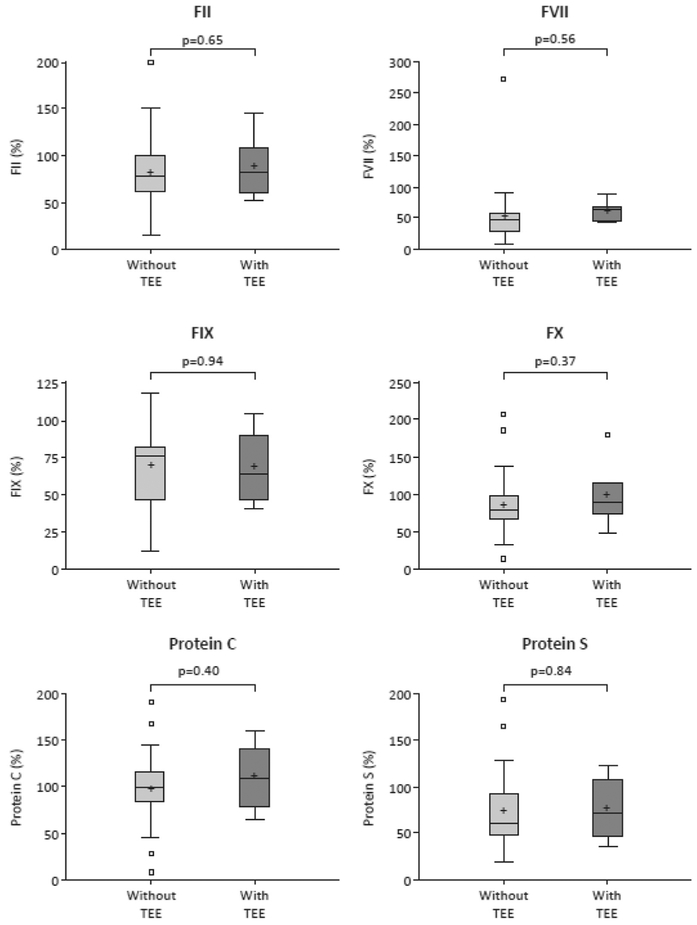
Median levels of coagulation factors and proteins C and S 0.5 hours after start of 4F-PCC infusion (integrated analysis). *F*, Factor. + Represents the mean of the data. The line in the middle of the box represents the median. The upper and lower bounds of the box are the 75th and 25th percentiles, respectively. The top of the whisker is the maximum (or 75th percentile+ [1.5×interquartile range] where outliers are present). The bottom of the whisker is the minimum (or 25th percentile–[1.5×interquartile range] where outliers are present).

We performed exploratory analyses to assess whether enhanced vitamin K–dependent coagulation factor levels were associated with thromboembolic events in this analysis population. The distributions of these factor levels were compared between patients with and without events in the 4F-PCC group (Figure E1, available online at http://www.annemergmed.com). After administration of 4F-PCC, we observed marked increases in coagulation factor levels, with median levels returning to physiologic range or below at 0.5 hours after the start of infusion.^14, 15^ We observed no notable differences in median levels of coagulation factors at 0.5 hours after start of 4F-PCC infusion in patients with and without an event (Figure E1, available online at http://www.annemergmed.com). Similar results were observed at 24 hours (data not shown). There was also no indication of an association between factor levels and events in the plasma group (data not shown).

Levels of proteins C and S were also markedly increased after 4F-PCC infusion.^14, 15^ We observed no significant differences in median proteins C and S levels between patients with and without thromboembolic events at 0.5 hours (Figure E1, available online at http://www.annemergmed.com) or 24 hours (data not shown) after start of 4F-PCC infusion. Results were similar for patients who received plasma (data not shown).

**Figure E2.**
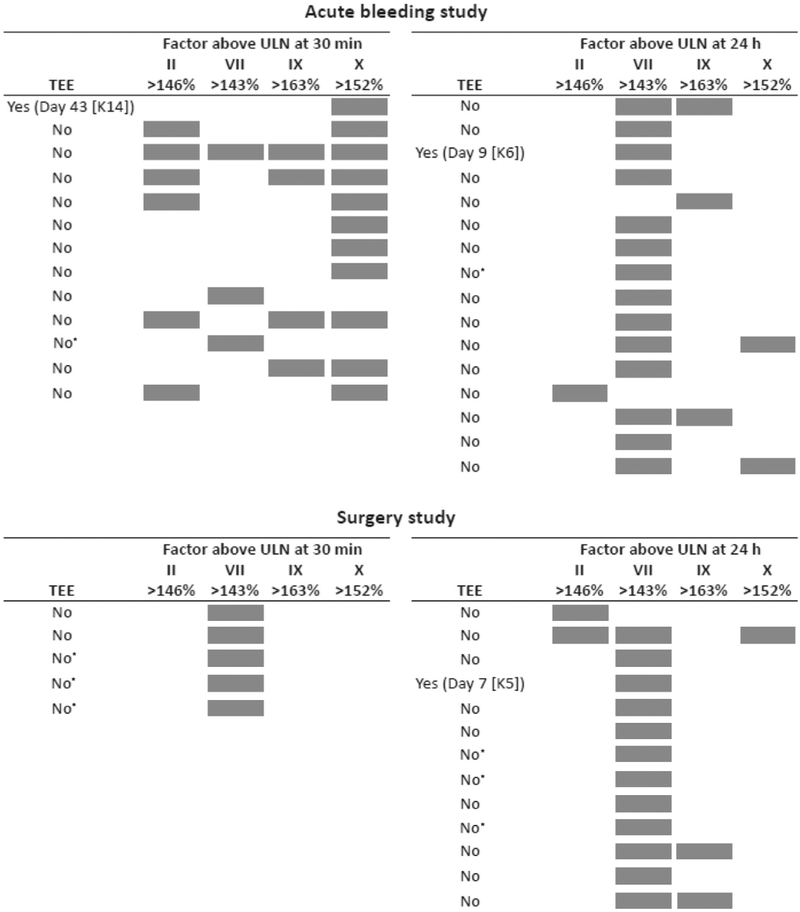
4F-PCC–treated patients with levels of at least 1 of the 4 vitamin K–dependent factors above the upper limit of normal in the acute bleeding and surgery studies. Normal adult reference range (95% range)^20^: FII=70% to 146%, FVII=67% to 143%, FIX=55% to 163%, FX=70% to 152%. *Patient had elevated FVII levels at 30 minutes and 24 hours postinfusion. *ULN*, Upper limit of normal.

We performed additional analyses to determine whether factor levels above the upper limit of normal were associated with events. In total, 43 of 191 patients (22.5%) in the 4F-PCC group had at least 1 factor above the upper limit of normal at 0.5 hours or 24 hours postinfusion (Figure E2, available online at http://www.annemergmed.com). However, only 3 of these 43 patients with factor levels above the upper limit of normal experienced events (patients K6 and K14 in the acute bleeding study and patient K5 in the surgery study) (Figure E2, available online at http://www.annemergmed.com).

## LIMITATIONS

We did not power the randomized controlled trials analyzed in this study to detect differences in safety outcomes such as thromboembolic events. In addition, we could not blind study staff and patients to treatment allocation because plasma and prothrombin complex concentrate have distinctly different appearances and administration procedures. However, a blinded safety adjudication board reviewed serious adverse events of interest, including thromboembolic events, increasing the robustness of this data set. The board evaluated only thromboembolic events deemed serious by unblinded investigators, meaning events investigators deemed nonserious did not receive independent blinded review; however, the data and safety monitoring board reviewed all events, serious and nonserious, whether deemed related or not by investigators. In addition, preoperative surgical patients may in some ways be pathophysiologically distinct from hemorrhaging patients; however, we provided patient-level data on all events, allowing readers to draw their own conclusions. Finally, we did not perform Doppler screening during or after the study period, so it is possible that subclinical asymptomatic thromboembolic disease was missed.

## DISCUSSION

Reversal of anticoagulant therapy with prothrombin complex concentrates may be associated with thromboembolic events.^13^ Reversing vitamin K antagonist anticoagulation with factor replacement by prothrombin complex concentrate or plasma, or even by vitamin K1 alone or by merely withdrawing vitamin K antagonist therapy, exposes patients to the underlying thromboembolic risk for which the vitamin K antagonist was initially prescribed. Our studies afford an opportunity to address some of the difficulties in characterizing thromboembolic event risk because they represent, to our knowledge, the largest controlled assessments of a 4F-PCC and plasma, in which patients were prospectively and rigorously followed for 45 days postinfusion (for serious adverse events). Post hoc analysis of combined safety data from these studies permitted a detailed examination of the effect of anticoagulation reversal strategies on thromboembolic events, allowing the characterization of type of event, timing, and clinical outcome to a degree of detail not previously reported.

The percentage of patients with thromboembolic events in each treatment group was similar (4F-PCC: 7.3%; plasma: 7.1%). Furthermore, the proportions of patients with treatment-related events (investigator assessed), serious events, and treatment-related serious events (assessed by the blinded safety adjudication board) in each group were also found to be similar. Although some of the cases were assessed as possibly related to study treatment (4F-PCC or plasma), most patients were not receiving anticoagulant therapy at the event, and such failure to resume anticoagulation in patients with existing thromboembolic risks can be considered a major contributor to thromboembolism.^16, 17^

We observed some potential differences between plasma and 4F-PCC with respect to the incidence of thromboembolic events subtypes. There were 8 vascular events in the 4F-PCC group (of which 4 were deep venous thromboses and none were pulmonary embolisms) versus 4 in the plasma group (including 1 deep venous thrombosis and 1 pulmonary embolism), but 1 cardiac event in the 4F-PCC group versus 6 in the plasma group. If the 2 data and safety monitoring board-referred, safety adjudication board–adjudicated cases of myocardial ischemia in the plasma group are removed from the analysis, the number of cardiac events observed in the plasma group remains higher than in the 4F-PCC group (4 versus 1). The increase in cardiac events in the plasma group might be due to increased blood volume after plasma transfusion, which would overwhelm cardiac output and may lead to ischemia. Although this suggests that appropriate monitoring may be required in plasma-treated patients with cardiac-related comorbidities, these results should be interpreted with caution owing to the small number of reported cases.

The increase in vitamin K-dependent factors as a result of study treatment could potentially influence the occurrence of thromboembolic events. However, in this analysis, there did not appear to be any consistent relationship between the timing of the events and the halflives of the administered factors. In addition, although the incidence of events was similar in both groups, events in the plasma group tended to cluster in the first week after study treatment, whereas events in the 4F-PCC group clustered a week later. It is plausible that the cluster of events that occurred on days 1 to 7 (11 and 5 in the plasma and 4F-PCC groups, respectively) is more likely to be related to the infused factors (and 10 of these 16 events were deemed at least possibly so by the investigators or the safety adjudication board) compared with events that occurred after day 7. Indeed, by the second week, factor levels would be more a reflection of the administered vitamin K and endogenous coagulation factor synthesis than of study product. Thus, it may be that the latter 15 events (11 and 4 in the 4F-PCC and plasma groups, respectively), which occurred 8 or more days after infusion (some even several weeks later), are more a consequence of preexisting thrombotic conditions rather than the infusion of study product per se. Nevertheless, unblended investigators deemed 7 events that occurred after week 1 (all in the 4F-PCC group) at least possibly related to the study treatment (Figure). Regardless of the cause of events, the observations of this study suggest that patients with high risk for thromboembolic events may benefit from resumption of vitamin K antagonist therapy as soon as the clinical circumstances allow. In patients with intracranial hemorrhage, the 2014 American Heart Association/American Stroke Association guidelines suggest resumption of anticoagulation at greater than or equal to 1 week.^18^

In terms of the potential relationship between vitamin K–dependent factors and thromboembolic events, patients who received a higher dose of study product were not more likely to have an event in this analysis. Furthermore, the lack of notable differences in median factor levels for patients with or without events indicates that the levels of the infused factors may not play a major role in determining events.

In conclusion, these results indicate that the use of 4F-PCC is not associated with an overall increased risk of thromboembolic events compared with plasma in patients requiring urgent anticoagulation reversal owing to major hemorrhage or before urgent surgical procedures. Differences in the number of thromboembolic event subtypes were observed between treatment groups, although the small number of events in each group limits data interpretation. Considering the age-related comorbidities and risk factors for thrombosis in this patient population, it is possible that events occur with a similar frequency irrespective of the reversal agent used.^16, 17^

## Figures and Tables

**Figure.**
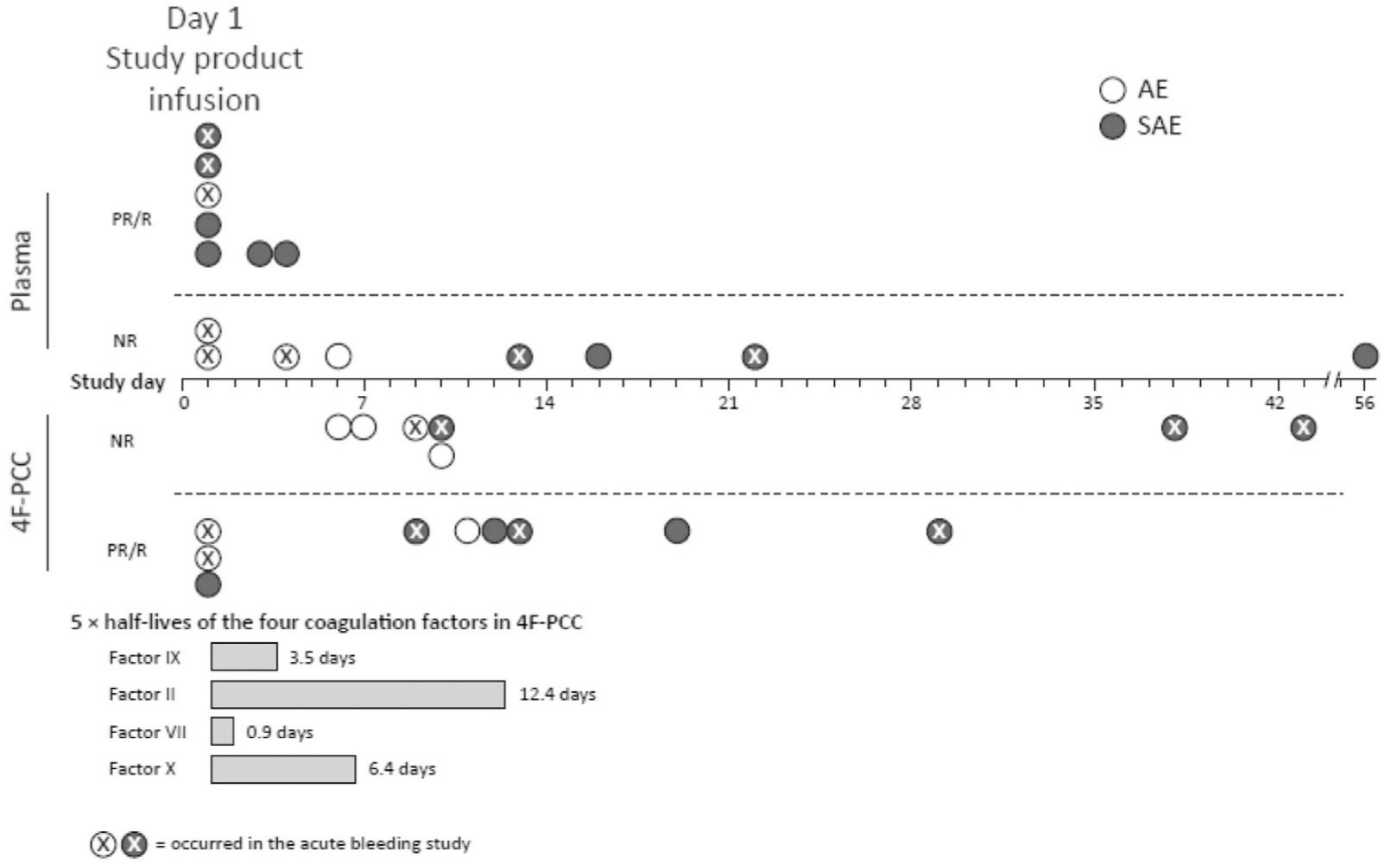
Thromboembolic events over time. Three SAEs were not confirmed as a thromboembolic events by the SAB; therefore, the SAB did not assess causality for these events (plasma group: PE [day 16]; 4F-PCC group: thrombosis [day 12] and MI [day 38]); causality indicated as assessed by investigators. *AE*, Adverse event; *PR*, possibly or probably related to the study product as assessed by investigators (for adverse events) or SAB (for SAEs); *R*, related to study product as assessed by investigators (for adverse events) or SAB (for SAEs); *NR*, not related to study product.

**Table 1.** Patients with thromboembolic events: selected demographics.

	Integrated Analysis	
Patients With TEEs	4F-PCC (n = 14)	Plasma (n = 14)
**Sex, No. (%)**		
Female	9 (64.3)	8 (57.1)
**Age, y**		
Mean (SD) [range]	75 (13) [51–90]	72 (11) [50–92]
≥70, No. (%)	9 (64.3)	9 (64.3)
Type of bleeding, No. (%)*		
GI/other nonvisible	4 (50.0)	5 (71.4)
Visible	1 (12.5)	0
ICH	3 (37.5)	0
Musculoskeletal	0	2 (28.6)
**Type of surgery/procedure, No. (%)**^†^		
Cranial neurosurgical	0	1 (14.3)
Cardiothoracic surgical	0	1 (14.3)
Major orthopedic surgical	2 (33.3)	3 (42.9)
Other surgical	2 (33.3)	0
Invasive procedure	2 (33.3)	2 (28.6)
**Reason for oral VKA therapy, No. (%)**		
Arrhythmia	9 (64.3)	10 (71.4)
Artificial heart valve or joint	0	1 (7.1)
Remote TEE	5 (35.7)	3 (21.4)
**Use of anticoagulant at TEE**	3 (21.4)	4 (28.6)

*TEE*, Thromboembolic event; *GI*, gastrointestinal; *ICH*, intracranial hemorrhage; *VKA*, vitamin K antagonist.

* For patients enrolled in the bleeding study (NCT00708435) only.

† For patients enrolled in the surgery study (NCT00803101) only.

**Table 2.** Details of patients with thromboembolic events (pooled safety population).

Patient	Study	Onset Day*	Age	Indication for VKA	Standardized Term	SAE	Causality^†^		Baseline INR	Death (Day)
							Investigator	SAB		
**4F-PCC**										
K1	Bleeding	1	66	Portal vein thrombosis	Thrombosis in device (fistula clot/thrombosis)	No	Yes	Not assessed	12.7	Yes (26)
K2	Bleeding	1	73	DVT	Venous thrombosis calf vein	No	Yes	Not assessed	5.4	No
K3	Surgery	1	64	Mitral valve replacement and AF	Ischemic stroke	Yes	Yes	Yes	2.7	No
K4	Surgery	6	78	DVT	Vena cava filter insertion^‡^	No	No	Not assessed	3.1	No
K5	Surgery	7	62	AF	Catheter-related complication (poor flow in dialysis catheter)	No	No	Not assessed	3.6	No
K6	Bleeding	9	54	DVT	Venous thrombosis limb (basilic vein clot at PICC line site)	No	No	Not assessed	9.2	No
K7	Bleeding	9	82	AF/St Jude’s valve	Ischemic stroke	Yes	No	Yes	4.4	No
K8	Bleeding	10	85	AF	Ischemic stroke	Yes	No	No	3.8	No
K9	Surgery	10	89	AF	CVA	No	No	Not assessed	2.2	No
K9	Surgery	11	As above	As above	Venous thrombosis limb (radial vein)	No	Yes	Not assessed	As above	As above
K10	Surgery	12	51	DVT	Microthrombosis of toes	Yes	Yes	Not confirmed as TEE	17	No
K11	Bleeding	13	80	AF	DVT right leg	Yes	Yes	Yes	2.2	No
K11	Bleeding	29	As above	As above	DVT left leg	Yes	No	Yes	As above	As above
K12	Surgery	19	90	AF	DVT	Yes	Yes	Yes	3.7	No
K13	Bleeding	38	88	AF	MI	Yes	No	Not confirmed as TEE	2.4	Yes (38)
K14	Bleeding	43	89	AF	Ischemic stroke	Yes	Yes	No	4.5	Yes (46)
**Plasma**										
P1	Bleeding	1	77	AF	Myocardial ischemia	Yes	Yes	Yes	2.0	No
P1	Bleeding	4	As above	As above	Thrombophlebitis	No	No	Not assessed	As above	No
P2	Bleeding	1	70	AF	Myocardial ischemia	Yes	Yes	Yes	3.1	No
P3	Bleeding	1	64	Aortic valve replacement	Acute MI	No	Yes	Not assessed	7.4	No
P4	Bleeding	1	75	AF	CVA	No	No	Not assessed	>3.99	No
P5	Bleeding	1	79	AF	Thrombosis in device (clotted NG tube)	No	No	Not assessed	2.8	No
P6	Surgery	1	75	DVT	Acute MI	Yes	No	Yes	5.9	No
P7	Surgery	1	59	DVT	DVT	Yes	Yes	Yes	2.1	No
P8	Surgery	2	85	AF	Embolic cerebral infarction	Yes	Yes	Yes	5.6	Yes (13)
P9	Surgery	4	61	AF	Acute MI	Yes	No	Yes	2.2	Yes (8)
P10	Surgery	6	50	AF	TIA	No	No	Not assessed	4.0	No
P11	Bleeding	13	68	AF	MI	Yes	No	No	2.5	No
P12	Surgery	16	92	AF	PE	Yes	No	Not confirmed as TEE	2.5	Yes (16)
P13	Bleeding	22	78	AF	CVA	Yes	No	No	3.0	No
P14	Surgery	56§	77	DVT	Acute CVA	Yes	No	No	2.1	No

*SAE*, Serious adverse event; *SAB*, Safety adjudication board; *INR*, international normalized ratio; *DVT*, deep venous thrombosis; *AF*, atrial fibrillation; *PICC*, peripheral into central circulation; *CVA*, cerebrovascular accident; *MI*, myocardial infarction; *NG*, nasogastric; *TIA*, transient ischemic attack; *PE*, pulmonary embolism.

* Onset study day of thromboembolic event, day of study product infusion is day 1.

† No: event deemed unrelated to the study product in the opinion of the investigator/SAB. Yes: event deemed at least possibly related to the study product in the opinion of the investigator/SAB. SAB evaluated only events that were SAEs and did not assess causality if the event was not confirmed as a thromboembolic one.

‡ The vena cava filter insertion (K4) was not considered a thromboembolic event by the lead author. The Food and Drug Administration assessed some of the adverse events and thromboembolic events differently from the study investigators/SAB, resulting in a difference in the numbers of thromboembolic events reported in the package insert for the 4F-PCC in the United States (Kcentra)^19^ compared with published study results.^14, 15^

§ Acute CVA was diagnosed on day 56 but included in the thromboembolic event analysis because of uncertain timing related to the start of the event.

**Table 3.** Characteristics of thromboembolic events.

	Integrated Analysis	
Thromboembolic Events (n = Events)	4F-PCC (n = 16)	Plasma (n = 15)
**Relatedness, investigator, No. (%)**		
Related	1 (6.2)	0
Possible	7 (43.8)	5 (33.3)
Not related	8 (50.0)	10 (66.7)
**Serious TEE, No. (%)**	9 (56.3)	10 (66.7)
Relatedness (investigator)	5 (31.3)	4 (26.7)
Relatedness (SAB)	5 (31.3)	6 (40.0)
Not confirmed as TEE (SAB)	2 (12.5)	1 (6.7)
**Type of event (by SOC), No. (%)**		
**Cardiac disorders**		
MI	1 (6.2)	1 (6.7)
Acute MI	0	3 (20.0)
Myocardial ischemia	0	2 (13.3)
**Nervous system disorders**		
Ischemic stroke	4 (25.0)	0
CVA	1 (6.2)	2 (13.3)
Embolic cerebral infarction	0	1 (6.7)
TIA	0	1 (6.7)
Acute CVA	0	1 (6.7)
**Vascular disorders**		
Thrombosis in device	1 (6.2)	1 (6.7)
Venous thrombosis limb	3 (18.8)	0
Thrombosis	1 (6.2)	0
DVT	3 (18.8)	1 (6.7)
Thrombophlebitis	0	1 (6.7)
PE	0	1 (6.7)
**Other**	2 (12.5)	0
**Severity of event by maximum intensity,No. (%)**		
Mild	2 (12.5)	5 (33.3)
Moderate	7 (43.8)	6 (40.0)
Severe	7 (43.8)	4 (26.7)
**Timing of event**		
Median, days	10	2
Mean, days	14	9
>1 wk postinfusion, No. (%)	4 (25.0)	11 (73.3)

*SOC*, System organ class.

**Table 4.** Comparison of characteristics between patients with thromboembolic events and the pooled study population.

	Integrated Analysis		
Variables	Patients With Events (n = 28)	Patients Without Events (n = 360)	Pooled Safety Population (N= 388)
**Age, y**			
Mean (SD) [range], y	73.6 (12.2) [50-92]	69 (13) [26-96]	69.3 (13.4) [26-96]
≥70, No. (%)	18 (64.3)	196 (54.4)	214 (55.2)
Type of bleeding, No. (%)*			
Gastrointestinal/other nonvisible	9 (32.1)	118 (32.8)	127 (32.7)
Visible	1 (3.6)	42 (11.7)	43 (11.1)
Intracranial hemorrhage	3 (10.7)	21 (5.8)	24 (6.2)
Musculoskeletal	2 (7.1)	16 (4.4)	18 (4.6)
**Type of surgery/procedure, No. (%)**^†^			
Cranial neurosurgical	1 (3.6)	1 (0.3)	2 (0.5)
Cardiothoracic surgical	1 (3.6)	4 (1.1)	5 (1.3)
Major orthopedic surgical	5 (17.9)	33 (9.2)	38 (9.8)
Other surgical	2 (7.1)	97 (26.9)	99 (25.5)
Invasive	4 (14.3)	28 (7.8)	32 (8.2)
**Reason for oral VKA therapy, No. (%)**			
Arrhythmia	19 (67.9)	175 (48.6)	194 (50.0)
Vascular disease	0	59 (16.4)	59 (15.2)
Artificial heart or joint	1 (3.6)	53 (14.7)	54 (13.9)
Remote thromboembolic event	8 (28.6)	63 (17.5)	71 (18.3)
Other	0	10 (2.8)	10 (2.6)

* For patients enrolled in the bleeding study (NCT00708435) only.

† For patients enrolled in the surgery study (NCT00803101) only.

## Editor’s Capsule Summary

### What is already known on this topic

Rapid reversal of warfarin anticoagulation provokes thromboembolic events in some patients. Pooled plasma is cheaper and more difficult to use; newer 4-factor prothrombin complex (4F-PCC) concentrates are more expensive and easier to deliver.

### What question this study addressed

Which causes more thromboembolic events and short-term mortality when reversing warfarin: pooled plasma or a 4F-PCC?

### What this study adds to our knowledge

In examination of data from 2 independent trials with 388 subjects, the frequency of thromboembolic events (7.1% and 7.3%) and mortality did not differ between pooled plasma and the newer concentrate.

### How this is relevant to clinical practice

Neither pooled plasma nor 4F-PCC produces a higher risk of new clot or death when used for rapid reversal.

## APPENDIX E1

Investigators identified AEs as possible thromboembolic events, using the following predefined standardized terms: “embolic and thromboembolic events, arterial” (eg, [acute] MI, ischemic stroke, and TIA); “embolic and thromboembolic events, venous” (eg, DVT, PE, thrombophlebitis, vena cava filter insertion, and venous thrombosis limb); and “embolic and thromboembolic events, vessel type unspecified and mixed arterial and venous” (eg, catheter-related complication, CVA, embolic cerebral infarction, thrombosis, and thrombosis in device).

The blinded SAB reviewed all serious thromboembolic events identified by the investigators on a case-by-case basis. The unblinded independent data and safety monitoring board could also refer cases to the SAB not coded as thromboembolic events but suspected as such. The study protocol dictated that in cases of disagreement between the unblinded investigator assessment and the blinded SAB assessment, the SAB determination would stand as final.

**Table E1.** Dose of study treatment per baseline international normalized ratio.

Baseline INR	Dose of 4F-PCC, IU of FIX/kg Body Weight*	Plasma, mL/kg Body Weight*
≥2-<4	25	10
≥4-≤6	35	12
>6	50	15

*FIX*, Factor IX.

* The dose calculation was based on 100-kg body weight for patients weighing greater than 100 kg. 4F-PCC was administered at an infusion rate of less than or equal to 3 IU/kg per minute. The plasma infusion rate was at the discretion of the clinical team in both the acute bleeding and surgery studies; however, in the acute bleeding study, an infusion rate of 1 unit per 0.5-hour interval was recommended by the protocol.

**Table E2.** Causality by event type.

Types of Disorders	4F-PCC				Plasma			
	Total Number of Events		Causality*		Total Number of Events		Causality*	
	AEs	SAEs	Investigator	SAB	AEs	SAEs	Investigator	SAB
Cardiac disorders	0	1	0	0	1	5	3	4
Nervous system disorders	1	4	2	2	2	3	1	1
Vascular disorders	4	4	6	3	2	2	1	1

* Causality indicates events at least possibly related to treatment in the opinion of the investigator/SAB. The investigator reviewed AEs and SAEs. The SAB only reviewed SAEs.

**Table E3.** Additional information on thromboembolic events.

Patient	Study	Onset Day*	Indication for VKA	Event Consistent With Indication for VKA†	Actual Dose, IU/kg	Receiving Anticoagulant at Event	Additional Risk Factors‡4
**4F-PCC**							
K1	Bleeding	1	Portal veinthrombosis	Yes	50.0	No	CAD, DM, HTN, end-stage renal disease, obesity
K2	Bleeding	1	DVT	Yes	35.0	No	AF, CHF, DM, HTN, CAD
K3	Surgery	1	Mitral valve replacement and AF	Yes	50.0 (overdose, should have been 25.0)	No	Cardiomyopathy, CHF, HTN, medication error, DVT left arm, atrial flutter
K4	Surgery	6	DVT	No	25.0	Yes	Metastatic prostate cancer
K5	Surgery	7	AF	No	25.0	No	End-stage renal disease, hemodialysis, HTN, CAD, stroke
K6	Bleeding	9	DVT	Yes	50.0	No	HTN
K7	Bleeding	9	AF/St Jude’s valve	Yes	35.0	No	Blood transfusions, CAD, CVA, HTN, MI
K8	Bleeding	10	AF	Yes	25.0	Yes	Blood transfusions, CHF, DVT/PE, thrombocytosis
K9	Surgery	10	AF	Yes	25.0	Yes	CAD, CHF, HTN, DM, atrial flutter, chronic renal insufficiency, chronic kidney disease stage 3
K9	Surgery	11	As above	No	As above	As above	As above
K10	Surgery	12	DVT	Yes	50.0	No	PE
K11	Bleeding	13	AF	No	25.0	No	CAD, HTN, immobility (multiple fractures), head trauma, MI
K11	Bleeding	29	As above	No	As above	No	As above
K12	Surgery	19	AF	No	25.0	No	Atrial flutter, PAF with RVR, sick sinus syndrome, HTN, right breast ductal carcinoma
K13	Bleeding	38	AF	No	25.0	No	Cardiomyopathy, CVA, DM, HTN
K14	Bleeding	43	AF	Yes	35.0	No	CAD, CHF, HTN, TIA
**Plasma**							
P1	Bleeding	1	AF	No	4.4	No	CAD, CHF and cardiomyopathy, EF of 27%, HTN, MI
P1	Bleeding	4	As above	No	As above	No	As above
P2	Bleeding	1	AF	No	6.0	No	CAD, CHF, cardiomyopathy, EF of 68%, CVA DM, HTN, ischemic colitis, SMA-occlusion hx
P3	Bleeding	1	Aortic valve replacement	No	13.5	No	CAD, CHF, DM, HTN, MI
P4	Bleeding	1	AF	Yes	17.7	No	HTN, DM
P5	Bleeding	1	AF	Yes	6.5	Yes	HTN, CAD, CHF, mitral valve incompetence, MI
P6	Surgery	1	DVT	No	12	No	Hypercoagulable state of unknown cause
P7	Surgery	1	DVT	Yes	7	No	HTN, PE, obesity
P8	Surgery	2	AF	Yes	11	No	CHF, HTN
P9	Surgery	4	AF	No	10	No	Severe AS, DM, obesity, EF 25%, hypotension
P10	Surgery	6	AF	Yes	12	Yes	CHF
P11	Bleeding	13	AF	No	9.9	Yes	CAD, stent placement
P12	Surgery	16	AF	No	10	No	CHF, immobility
P13	Bleeding	22	AF	Yes	10.0	Yes	DM, ARF and CRF, CHF, HTN, obesity
P14	Surgery	56^§^	DVT	No	10	No	Intermittent irregular heart rhythm, HTN, CAD, DM

*CAD*, Coronary artery disease; *DM*, diabetes mellitus; *HTN*, hypertension; *CHF*, congestive heart failure; *PAF*, paroxysmal atrial fibrillation; *RVR*, rapid ventricular response; *EF*, ejection fraction; *SMA*, superior mesenteric artery; *hx*, history; *AS*, aortic stenosis; *ARF*, acute renal failure; *CRF*, chronic renal failure.

The vena cava filter insertion (K4) was not considered a thromboembolic event by the lead author. The Food and Drug Administration assessed some of the AEs and thromboembolic events differently from the study investigators/SAB, resulting in a difference in the numbers of thromboembolic events reported in the package insert for the 4F-PCC in the United States (Kcentra)^19^ compared with published study results.^14, 15^

* Onset study day of thromboembolic event, day of study product infusion is day 1.

† Thromboembolic event considered consistent with the patient’s original VKA indication by the lead author.

‡ In addition to indication for VKA treatment.

§ Acute CVA was diagnosed on day 56 but included in the thromboembolic event analysis because of uncertain timing related to the start of the event.
